# Peripheral superior cluneal nerve stimulation for intractable low back pain: Combined fluoroscopy and ultrasound technique, A case series

**DOI:** 10.1016/j.inpm.2025.100542

**Published:** 2025-02-06

**Authors:** Nicolas M. Mas D Alessandro, Faria Nisar, Hesham Elsharkawy

**Affiliations:** a The MetroHealth System, Case Western Reserve University School of Medicine, United States; b Department of Anesthesiology and Perioperative Services, MetroHealth System, 2500 MetroHealth Drive, Cleveland, OH, 44109, United States; c Department of Anesthesiology, Pain, and Healing Center, MetroHealth System, United States; d Outcomes Research Consortium, Houston, TX, United States

**Keywords:** Peripheral nerve stimulation, Cluneal neuralgia, Chronic low back pain

## Abstract

**Background:**

Chronic low back pain (CLBP) is a common and debilitating condition often difficult to diagnose, with entrapment of the superior cluneal nerves (SCN) being a overlooked cause. Cluneal neuralgia (CN) arises from injury to the SCN and can significantly impact patients' quality of life.

**Objectives:**

This case series aims to evaluate the effectiveness of the Micro Lead - SPRINT Peripheral Nerve Stimulation (PNS) System, (Cleveland, Ohio, USA) for treating cluneal neuralgia, utilizing both fluoroscopic and ultrasound guidance for accurate nerve localization.

**Methods:**

A retrospective review was conducted on six nonconsecutive patients who underwent Micro Lead - SPRINT Peripheral Nerve Stimulation (PNS) System, (Cleveland, Ohio, USA) implantation for cluneal nerve entrapment at MetroHealth System between August 2021 and January 2024. Patient selection focused on individuals with cluneal neuralgia refractory to conservative treatments. Data collection included demographics, pain characteristics, opioid usage, and follow-up evaluations at 30, 60, 90 days, and 2 years post-procedure. Outcomes were assessed using the Numerical Rating Scale (NRS) for pain. Dividing the NRS score by the maximum score (10) and multiplying by 100 expresses pain intensity as a percentage.

**Results:**

Among the six nonconsecutive patients (83 % female, mean age 60 years), the mean pain score prior to implantation was 7.1. At follow-up, five patients reported over 50 % improvement in pain and functional status. Three patients with prior opioid use had varying outcomes regarding opioid consumption post-procedure.

**Conclusion:**

The Micro Lead - SPRINT Peripheral Nerve Stimulation (PNS) System, (Cleveland, Ohio, USA) demonstrates promise as an effective treatment for cluneal neuralgia, leading to reductions in pain and improvements in daily living activities. Further studies are warranted to validate these findings.

## 1 Introduction

Chronic low back pain is common and debilitating, with oftencomplex and challenging diagnoses [1]. A frequently overlooked cause of low back and buttock pain is cluneal neuralgia (CN) [2]. CN can result from injury to any of the three sets of cluneal nerves: superior, middle, or inferior. The superior cluneal nerves (SCN) consist of three main branches: medial, intermediate, and lateral [3].

The SCN arise from the dorsal rami of the lower thoracic and lumbar nerve roots (L1-L3) through their lateral cutaneous branches. It has a diameter of about 1.1 mm and courses superficially in the subcutaneous tissue of the lower back above the lumbar muscles [2]. As the nerve roots exit the spine, they pass through the psoas major and erector spinae muscles, traveling posteriorly to the quadratus lumborum muscle through the thoracolumbar fascia, eventually reaching the iliac crest [1].

An entrapped SCN can cause chronic, unilateral low back pain, typically between the lower ribs and buttocks, sometimes radiating to the buttock, thigh, or groin. The pain, described as sharp, stabbing, or burning, worsens with bending, rotating, or prolonged sitting [4,5].

Treatment for CN often requires a multidisciplinary approach. Options range from conservative management to more advanced interventions like peripheral nerve stimulation (PNS) and surgery. Initial treatment generally focuses on conservative measures, including physical therapy, NSAIDs, and local anesthetic or corticosteroid injections [6].

Temporary percutaneous PNS, a minimally invasive technique, is gaining popularity for its ease of electrode placement. It is typically considered when conservative measures fail to provide relief [7].

In this case series, we will describe our experience in implanting the Micro Lead -SPRINT PNS System- (SPR) Therapeutics, Inc., (Cleveland, Ohio, USA), for superior cluneal neuralgia. The primary advantage of our case series lies in the utilization of both X-ray and ultrasound for nerve localization.

## 2 Methods

### 2.1 Study design and population

This study retrospectively reviewed data from six nonconsecutive patients who underwent implantation of the Micro Lead - SPRINT PNS System, Ohio, US for the treatment of cluneal nerve entrapment at MetroHealth System between August 2021 and January 2024. Institutional Review Board (IRB) exemption was obtained for this review. The SPRINT PNS device, which is FDA-approved for temporary use for up to 60 days, was activated and used during this period.

### 2.2 Patient selection

Patients were selected based on a clinical presentation of chronic pain attributed to cluneal nerve pain, which was refractory to conservative treatments including physical therapy, medications, and local nerve blocks. All patients had persistent pain despite these interventions, making them candidates for peripheral nerve stimulation (PNS).

Diagnostic criteria: tenderness over the posterior iliac crest, and positive responses to diagnostic nerve blocks. Lumbar MRI ruled out other conditions.

### 2.3 Procedure details

The PNS system was implanted using a percutaneous technique under a combined approach with fluoroscopy and ultrasound guidance.

### 2.4 Technique

**Fig. 1:**
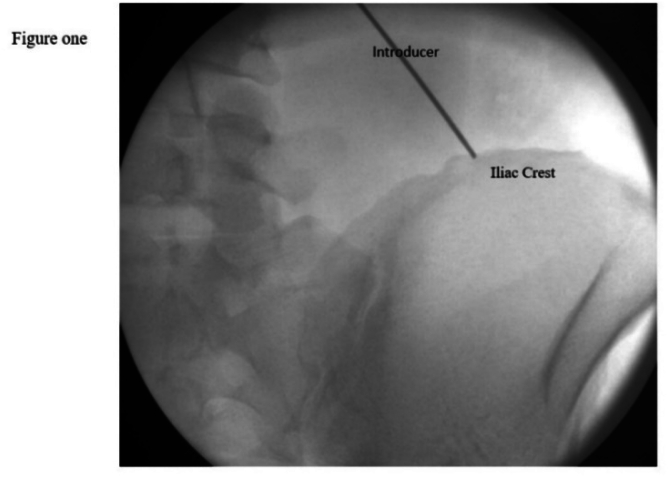
Xray AP View *Shows the introducer direction from medial cranial to caudal lateral with the tip above the iliac crest.*

**Fig. 2:**
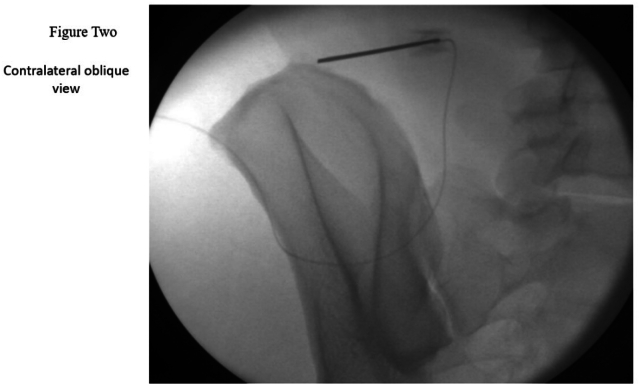
Xray Contralateral oblique view; *The introducer is shown in a contralateral oblique view, with its tip positioned above the iliac crest.*

**Fig. 3:**
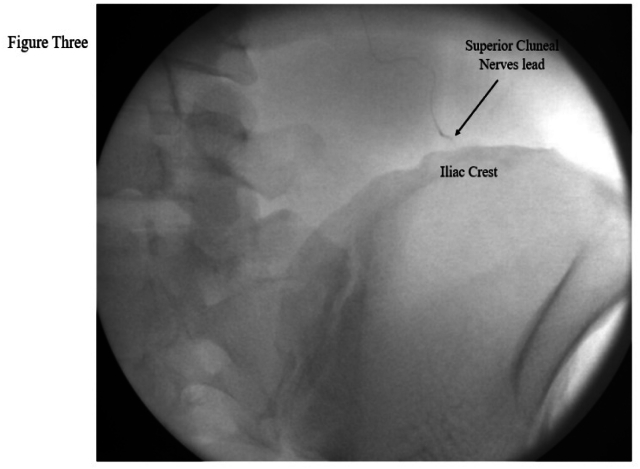
Xray AP View *Shows the lead location.*

1. In an AP X-ray view, the iliac crest was identified. The tip of the lead was positioned approximately 7 cm lateral from the midline and ½ to 1 cm above the middle third of the iliac crest.
2. Ultrasound was used to locate the iliocostalis part of the erector spinae muscle (ESP), the posterolateral aspect of the quadratus lumborum muscle, the medial aspect of the latissimus dorsi muscle, and the iliac crest lateral to L4 transverse process (TP). For low-BMI patients, a high-frequency probe was used, while a low-frequency probe was used for high-BMI patients. The ultrasound was oriented in an oblique view with the probe positioned medially cranial and laterally caudal, aligned with the introducer. The ultrasound machine used was a GE Venue 50. Approximately 4–6 cm medial and cranial to the lead tip location, the skin and subcutaneous tissue were infiltrated with 1 % lidocaine.
3. A 20-gauge blunt introducer was advanced with ultrasound and fluoroscopy guidance in the AP and contra lateral oblique views. The introducer tip was tracked on ultrasound, in-plane parallel approach positioned superficial to the quadratus lumborum (QL) and lateral to the iliocostalis muscle (Fig. 1, Fig. 2). The approach was from medial cranial to caudal lateral (Fig. 4). Proper placement was confirmed by the patient's feedback, who reported a comfortable vibration sensation in the targeted nerve distribution area (Fig. 3). Then the introducer stylet was removed, and the preloaded lead was advanced through the introducer cannula, deploying the lead by applying gentle pressure over the needle tip and lead location.

**Fig. 4:**
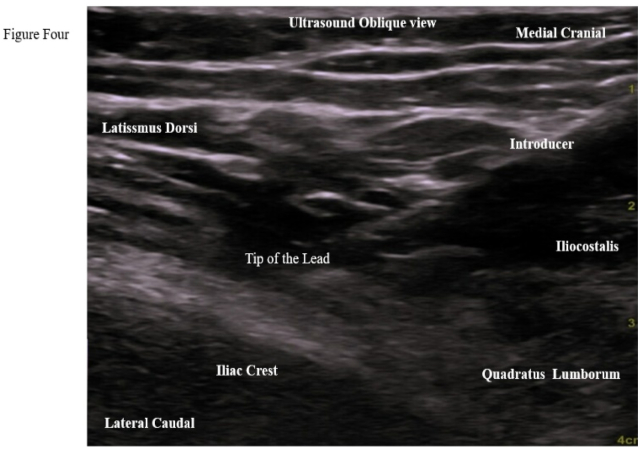
Ultrasound image showing the in-plane approach, with the tip of the introducer and the lead positioned lateral to the iliocostalis, between the latissimus dorsi and quadratus lumborum muscles.
4. Final placement was verified with AP and lateral fluoroscopy views above the middle third of the iliac crest, and with ultrasound confirming the lead tip in the lateral third of the erector spinae muscle, just cranial to the iliac crest.
5. In cases where bilateral leads were used, both leads exited medially, positioned just lateral to the spinous process. This setup enabled the use of a single dressing to cover both leads.

### 2.5 Data collection

Patient data was collected retrospectively from the notes of patient encounters within the electronic medical records, including demographics, pain characteristics, opioid usage, and medical history. Clinical developments were tracked at 30, 60, 90 days, and 2 years post-procedure.

### 2.6 Outcome measures

1. Pain Scores: Patients' pain levels were evaluated using the Numerical Rating Scale (NRS) at various follow-up intervals (30, 60, 90 days, and 2 years). A reduction in NRS scores of more than 5 was considered a successful outcome. In addition, percentage pain reduction was calculated to provide further insight into the patient's progress, using the formula: (NRS score at follow-up/baseline NRS score) × 100. This calculation was performed to complement the numerical reduction and offer a clearer measure of pain improvement over time.
2. Improvements in Daily Living: Patients' improvements in daily activities, including dressing, walking, and sleeping, were collected.
3. Opioid Usage: The reduction in opioid consumption post-procedure was reported regardless of whether the patient received an opioid prescription during the following appointment.
4. Complications.

## 3 Results

A total of six nonconsecutive patients (83 % female, mean age 60 years) with chronic superior cluneal pain underwent temporary peripheral nerve stimulator implantation targeting the superior cluneal nerves. The mean duration of symptoms before PNS implantation was 18 months. All patients had tried various treatments with no or limited success, including topical and oral medications, physical therapy, acupuncture, and one patient had received various cortisone conservative injections. Imaging studies excluded significant lumbar spine degenerative changes and neural foraminal stenosis. The patients had associated diagnoses of depression in three of the six cases (cases 1, 2, and 6). Four patients had bilateral leads, and two patients had unilateral leads. Notably, one patient received an L2 medial branch PNS in addition to a single lead cluneal PNS.

Pre-implantation, the mean pain score among patients (measured on a visual analog scale [VAS] of 0–10) was 7.1. The mean duration of the implant was 61 days, with only one patient having the implant for one week and two patients having the implant for more than 75 days (see Table 1).

**Table 1:** Patient characteristics.

Patient ID^a^	Gender	Age (years)	BMI (kg/m^2^)	Duration of Implant (days)	Leads
1	F	61	30.42	64	bilateral
2	F	68	40.4	64	bilateral
3	F	62	29.29	48	bilateral
4	F	68	25	30	unilateral
5	F	66	23.24	8	unilateral
6	M	39	36.22	85	bilateral

a “Patient ID” refers to a unique identifier used solely for the purpose of maintaining patient confidentiality and does not hold any additional significance.

The patients did not consistently attend follow-up visits, which limited ongoing monitoring. However, we were still able to observe improvements in their comfort during daily activities (such as dressing, walking, and sleeping) and a reduction in pain over time.

Although not measured using a specific scale, patients reported subjective improvements in activities, sleep, and walking, with five patients noting more than a 50 % improvement in functional status at the 30-day follow-up. Of the total of three patients who were consuming opioids before the intervention, two continued to require opioid therapy, and one patient stopped opioid therapy after the PNS.

**Fig. 5:**
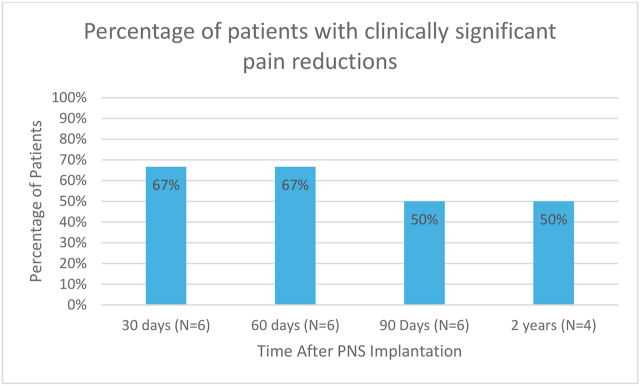
Percentage of patients with clinically significant pain reductions.

Fig. 5, illustrates the percentage of patients experiencing clinically significant pain reduction at various time points following peripheral nerve stimulation (PNS) implantation. At both 30 and 60 days post-implantation, 67 % of patients reported significant pain relief. However, this percentage decreased to 50 % at 90 days and remained stable at 50 % at the 2-year follow-up.

**Fig. 6:**
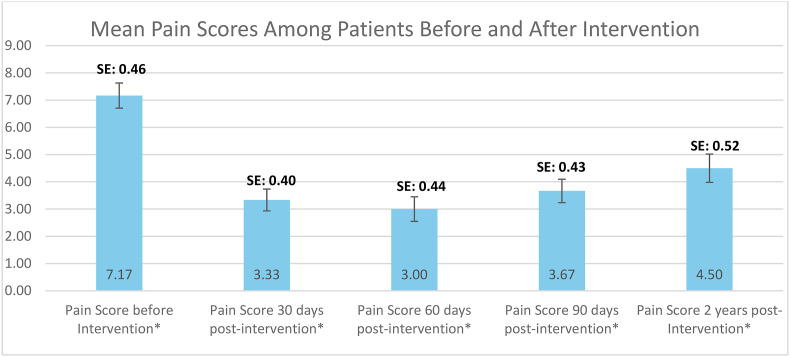
Mean pain scores among patients before and after intervention.

Fig. 6. The bar graph shows the progression of mean pain scores before and after the intervention. Pain levels decreased from a baseline of 7.17 to 3.33 at 30 days post-intervention and further to 3.00 at 60 days. By 90 days, the score slightly increased to 3.67, and at the two-year follow-up, it reached 4.50. While there was a gradual increase in pain scores over time, they remained markedly lower compared to baseline. The error bars indicate variability in the measurements, supporting the consistency of the observed trend. These findings suggest sustained, though slightly diminishing, pain relief over the two-year period (see Table 2).

Table 3 shows pain scores of six patients before and after intervention at multiple time points (30, 60, 90 days, and 2 years).

**Table 2:** Pain Management before and after implant.

Patient ID^a^	History of Opioid Use	Opioid Use		Pain Management Changes	Pain Score (NRS)	
		Pre-procedure	Post-Procedure		Pre-Procedure	Post-procedure^b^
1	Yes	No	No	None	8	4
2	Yes	Yes	Yes	None	10	2
3	Yes	Yes	No	Stop meloxicam, oxycodone	7	1
4	No	No	No	Stop lidocaine patch	9	8
5	No	Yes	Yes	Add meloxicam	2	2
6	No	No	No	None	7	1

a “Patient ID” refers to a unique identifier used solely for the purpose of maintaining patient confidentiality and does not hold any additional significance.

b We consider post procedure a 60 days follow up. The Tble 5 shows pain scores of six patients before and after intervention at multiple time points (30, 60, 90 days, and 2 years).

**Table 3:** Numerical pain scores.

Patient ID∗∗	Pain Score before Intervention∗	Pain Score 30 days post-intervention∗	Pain Score 60 days post-intervention∗	Pain Score 90 days post-intervention∗	Pain Score 2 years post-Intervention∗
1	8	4	4	4	4
2	10	5	2	5	5
3	7	1	1	2	∗∗∗
4	9	7	8	8	∗∗∗
5	2	2	2	2	2
6	7	1	1	1	7

∗ Numerical Rating Scale (NRS)

∗∗ “Patient ID” refers to a unique identifier used solely for the purpose of maintaining patient confidentiality and does not hold any additional significance

∗∗∗ Not applicable; no information recorded

Analyzing each case in detail:

P1: The patient presented one month after the trial procedure with Depomedrol plus Bupivacaine injection, reporting 80 % pain relief. Ten months later, the patient returned for the placement of the peripheral nerve stimulation (PNS).

During the initial consultation at 30 days, the patient reported using the device for more than 12 h, leading to an improvement in daily activities. The patient sometimes felt contractions but found them comfortable. However, at the two-month follow-up, the patient presented with improvement in her low back pain but with a local infection, which was successfully treated with cephalexin. At a 2-year follow-up, the patient reported no improvement in pain.

P2: At 30 days follow up there was 50 % improvement. The patient returned two months after the procedure, reporting over 80 % improvement in lower back pain, walking, and sleep. At the three-month evaluation post-implant, the patient still had a significant improvement in pain, more than 50 %. However, at two years follow up they still experienced some heaviness and tightness.

P3: The patient returned two months after the trial procedure with Depomedrol plus Bupivacaine, reporting 70 % pain relief. The patient was more active, satisfied, and had improved her activity level. Therefore, two months later, the PNS was implanted, resulting in 90 % pain relief with improvement in sleep, increased activity, and improved walking at 30 days follow up and 60 days follow up. At the three-month evaluation post-implant, the patient still had a significant improvement in pain, more than 50 %. At a 2-year follow-up, the patient did not want to reveal her pain status.

P4: This patient presented with lumbar axial pain, supported by MRI evidence of severe multifidus atrophy and positive physical findings. The patient underwent a single lead placement PNS at L3 for the L2 medial branch of the dorsal primary ramus. Seven days later, there was an improvement in pain, with the patient using the device nearly 24/7. The patient experienced significant improvement. One month later, a one-lead PNS was placed at the Right Superior Cluneal area. There was an improvement in pain, although it persisted, so high frequency was continued for two more weeks. One month after the second PNS placement, both were removed. The patient reported a 20–30 % improvement in pain in the buttock and hip area. At the two-month follow-up after the second PNS was removed, the patient presented with an area sensitive to touch. The patient experienced pain relief along with an improvement in her activity level. During this visit, the patient also reported new shoulder pain. At the three-month evaluation post-implant, the patient still experienced pain, and developed a new pain condition. At a 2-year follow-up, the patient was unable to be contacted.

P5: The patient returned one-week post-procedure requesting the removal of the PNS. She was informed that a 60-day evaluation period was necessary, but she still requested removal. The patient was uncomfortable with the external pulse generators, the logistics of handling them, and the dressing.

P6: The evaluation was conducted with an 85-day (3-month) interval between the intervention and the subsequent consultation. The patient reported that the leads came out during this interval. Upon returning to the consultation, the patient now had brachial pain but no pain at the cluneal level, with 75–100 % pain relief at the cluneal level. The patient experienced pain relief along with an improvement in her activity level. The patient detailed that this response persisted from when the leads came out until five months later. During the consultation five months after the procedure, the patient presented with multiple pains at other levels and in the lower back, with the return of pain. At a 2-year follow-up, the patient revealed that although the pain improved at the beginning, now it is back, and would like to have the PNS again.

## 4 Discussion

Our results suggest that SCN PNS implantation may lead to reduced pain intensity and improved functional status in some patients with chronic superior cluneal nerve pain.

Five out of six patients experienced an improvement in activities of daily living, accompanied by a reduction of at least 3 points on the pain scale after two years of follow-up. These findings indicate a potential for PNS to enhance the ability to perform daily activities, although individual responses may vary. The safety profile appears acceptable with minimal adverse events reported, primarily local site infections.

Some patients presented with pain in other areas post-intervention. It is important to clarify that post-intervention outcomes were specifically obtained for the area of pain targeted by the peripheral nerve stimulation (PNS). Many patients have other sources of pain or pain generators that can affect measurements of functional status and pain scores. By concentrating on the specific area where the PNS was inserted, we can more accurately assess the effectiveness of the intervention and avoid confounding results from other pain sources. This focused approach provided a clearer understanding of the true impact of PNS on the targeted pain area.

After analyzing all our data from the medical records, we tried to contact the patients, and two of the six patients could not be contacted for a two year follow-up. Interestingly, one patient reported no change in pain, remaining in a critical condition and unable to mobilize, emphasizing how critical the pain score in this patient was. A second patient, despite experiencing pain and expressing a desire to undergo the procedure again, was able to perform daily activities without any problems. However, this patient reported that their sleep quality was impaired.

PNS can be a valuable treatment option for neuropathic pain in the short term, particularly in patients who have failed conventional therapies. However, further research is needed to better understand the optimal patient selection criteria, long-term efficacy, and cost-effectiveness of PNS compared to other treatment modalities. These conclusions are in concordance with the current literature [[8], [9], [10]].

Regarding the technique, incorporating ultrasound enhanced the accuracy of localization of the superior cluneal nerves on the lateral superficial aspect of the iliocostalis and quadratus lumborum muscles. Ultrasound determined both the depth and precise location of the nerves, eliminating the need for the contralateral oblique X-ray view to stay above the iliac crest. We still used the contralateral oblique view as a confirmatory method. This approach potentially reduced the risk of X-ray exposure.

In summary, X-ray was employed to identify the iliac crest, while ultrasound was utilized to identify the tissue planes adjacent to the iliocostalis part of the erector spinae and quadratus lumborum muscles. All superior cluneal nerves emerged superficial to the lateral half of the iliocostalis muscle, corresponding to the lateral third of the entire erector spinae muscle in the lumbar region. This anatomical feature makes this approach ideal for covering the desired region without the need for additional X-ray radiation.

Previous investigations had attempted this selective technique on cadavers to test its feasibility before applying it to healthy individuals. In these studies, a superior cluneal nerve block was combined with transversalis fascia plane and subcostal nerve blocks using ultrasound guidance, allowing precise localization of the superior cluneal nerve (SCN) [11].

## 5 Conclusion

Temporary PNS shows promise as a treatment option for superior cluneal neuralgia showing a decrease in pain scores as well as functional outcomes. Furthermore, the use of the combined ultrasound and Xray technique potentially can enhance the accuracy of localization and allow less X-ray exposure.

## Financial and non-financial competing interests

Hesham Elsharkawy is consulting for SPR and Curonix and have stock options in Neuronoff. The rest of the authors declare that they have no competing interests.

## Statement and declarations

Dr. Elsharkawy is consultant of SPR Therapeutics.

## Funding statement

This project was supported in part by the Clinical and Translational Science Collaborative (CTSC) of Cleveland which is funded by the National Institutes of Health (NIH), National Center for Advancing Translational Science (NCATS), Clinical and Translational Science Award (CTSA) grant, UL1TR002548. The content is solely the responsibility of the authors and does not necessarily represent the official views of the NIH. If there are other authors, they declare that they have no known competing financial interests or personal relationships that could have appeared to influence the work reported in this paper.

## Funding statement

This project was supported in part by the 10.13039/100012729Clinical and Translational Science Collaborative (CTSC) of Cleveland which is funded by the 10.13039/100000002National Institutes of Health (NIH), 10.13039/100006108National Center for Advancing Translational Science (NCATS), Clinical and Translational Science Award (CTSA) grant, UL1TR002548. The content is solely the responsibility of the authors and does not necessarily represent the official views of the NIH.

## Declaration of competing interest

The authors declare the following financial interests/personal relationships which may be considered as potential competing interests: Nicolas Mario Mas D Alessandro reports administrative support and statistical analysis were provided by MetroHealth Medical Center. Hesham Elsharkawy reports a relationship with SPR Therapeutics Inc that includes: consulting or advisory. Human subjects: Consent was obtained or waived by all participants in this study. STUDY00000243: Peripheral Nerve Stimulator issued approval he MetroHealth IRB Federalwide Assurance (FWA) number 00003938. On July 5, 2024, the IRB approved the above submission using an expedited review procedure in accordance with 45 CFR 46.110(b). Animal subjects: All authors have confirmed that this study did not involve animal subjects or tissue. Conflicts of interest: In compliance with the ICMJE uniform disclosure form, all authors declare the following: Payment/services info: All authors have declared that no financial support was received from any organization for the submitted work. Financial relationships: All authors have declared that they have no financial relationships at present or within the previous three years with any organizations that might have an interest in the submitted work. Other relationships: Hesham Elsharkawy is consulting for SPR and Curonix and have stock options in Neuronoff.
